# Application of Random Forest and data integration identifies three dysregulated genes and enrichment of Central Carbon Metabolism pathway in Oral Cancer

**DOI:** 10.1186/s12885-020-07709-0

**Published:** 2020-12-14

**Authors:** Srija Mukhopadhyay, Sahana Ghosh, Debodipta Das, P. Arun, Bidyut Roy, Nidhan K. Biswas, Arindam Maitra, Partha P. Majumder

**Affiliations:** 1 National Institute of Biomedical Genomics, Kalyani, 741251 India; 2 Tata Medical Centre, Kolkata, India; 3 Indian Statistical Institute, Kolkata, India

**Keywords:** Random Forest, Epigenomic, Transcriptomic, Integrative analysis, Gingivo-buccal oral cancer

## Abstract

**Background:**

Studies of epigenomic alterations associated with diseases primarily focus on methylation profiles of promoter regions of genes, but not of other genomic regions. In our past work (Das et al. 2019) on patients suffering from gingivo-buccal oral cancer – the most prevalent form of cancer among males in India – we have also focused on promoter methylation changes and resultant impact on transcription profiles. Here, we have investigated alterations in non-promoter (gene-body) methylation profiles and have carried out an integrative analysis of gene-body methylation and transcriptomic data of oral cancer patients.

**Methods:**

Tumor and adjacent normal tissue samples were collected from 40 patients. Data on methylation in the non-promoter (gene-body) regions of genes and transcriptome profiles were generated and analyzed. Because of high dimensionality and highly correlated nature of these data, we have used Random Forest (RF) and other data-analytical methods.

**Results:**

Integrative analysis of non-promoter methylation and transcriptome data revealed significant methylation-driven alterations in some genes that also significantly impact on their transcription levels. These changes result in enrichment of the Central Carbon Metabolism (CCM) pathway, primarily by dysregulation of (a) *NTRK3*, which plays a dual role as an oncogene and a tumor suppressor; (b) *SLC7A5* (*LAT1*) which is a transporter dedicated to essential amino acids, and is overexpressed in cancer cells to meet the increased demand for nutrients that include glucose and essential amino acids; and, (c) *EGFR* which has been earlier implicated in progression, recurrence, and stemness of oral cancer, but we provide evidence of epigenetic impact on overexpression of this gene for the first time.

**Conclusions:**

In rapidly dividing cancer cells, metabolic reprogramming from normal cells takes place to enable enhanced proliferation. Here, we have identified that among oral cancer patients, genes in the CCM pathway – that plays a fundamental role in metabolic reprogramming – are significantly dysregulated because of perturbation of methylation in non-promoter regions of the genome. This result compliments our previous result that perturbation of promoter methylation results in significant changes in key genes that regulate the feedback process of DNA methylation for the maintenance of normal cell division.

**Supplementary Information:**

The online version contains supplementary material available at 10.1186/s12885-020-07709-0.

## Background

For various cancers both DNA methylation and gene expression data have been analyzed separately and alterations have been found to be associated with susceptibility and outcome [1, 2]. It is well known that DNA methylation impacts on gene expression. Therefore, attempts have been made to perform integrative analyses of these two types of data to draw robust inferences [3]. Various methods of data integration have been used [4, 5]. Methylation and expression data are high volume, highly correlated data. Further, the number of genes or DNA regions/sites on which data are collected are orders of magnitude higher than the number of patients and controls. This is commonly known as the “large *p*, small *n*” problem or “curse of dimensionality” in Statistics. Many statistical methods involve inversion of a matrix for obtaining estimates of parameters. When the number of variables (p), on which data are available, for each patient exceeds the total number of patients (n), inversion of the relevant matrix becomes impossible [6]. This results in parameter estimates that are not unique; therefore, inferences are liable to be compromised. Random forest (RF) is a machine learning inferential method that is data-adaptive and tree-based. It handles correlated and large data sets very efficiently and is, therefore particularly appealing for analysis of high-dimensional genome data. Normally, only a small portion of a high-dimensional data is associated with a phenotype. A regression framework does not apply to this scenario. The highly correlated nature of genomic data also makes the application of standard statistical models inappropriate. RF is a non-parametric tree-based approach that is particularly suited for such data-analysis problems. RF can also be used to select and rank variables by taking advantage of variable importance measures. A good review of RF in genomic data analysis can be found [7].

We have used RF methodology to identify gene-body methylation differences between tumor and adjacent normal tissues in patients with oral squamous cell carcinoma of the gingivo-buccal region (OSCC-GB), the most common form of oral cancer in India [8, 9]. We then integrated the knowledge thus obtained with data on levels of transcription of genes, which we use as a proxy for gene-expression levels, to discover methylation-driven alterations in the gene-body regions of the genome that significantly associate with dysregulation of genes in oral cancer.

DNA methylation occurs predominantly on cytosines followed by guanine residues (CpG). This type of methylation is referred to as CpG methylation. Although about 3–4% of all cytosines are methylated in normal human DNA, there are CpG islands,which are clusters of CpG dinucleotides in GC-rich regions, that remain unmethylated in all normal tissues [10]. Normally, a gene is transcribed if the CpG island in the promoter region remains unmethylated. But in cancer, the transcription of a tumor suppressor gene is silenced by the methylation of promoter CpG island of that gene. We had earlier analyzed data on methylation in CpG sites in the known promoter regions of all genes, but ignored gene-body CpG sites; sites that are on the coding regions of genes [4]. In our previous study, we identified about 200 genes that showed significant inverse correlation between promoter methylation and expression. These included a set of genes that act as transcription factors and genes associated with multiple cancer types. A significant finding of the study [4] was the identification of significant upregulation of CD274 and CD80 via promoter hypomethylation and hence immunosuppressive effects in OSCC-GB. Since in our previous study we had not considered gene body methylation, in the present study we have applied a modern data-adaptive method (RF) on gene-body methylation data and subsequently integrated with gene expression data. Our present analysis has resulted in the identification of some dysregulated genes and a pathway that were not identified in our earlier [4] analysis of promoter methylation and expression.

## Methods

### Patient recruitment and sample collection

This study was approved by the Institutional Ethics Committees of the Tata Medical Centre and the National Institute of Biomedical Genomics, India. Patients suffering from oral squamous cell carcinoma of the gingivobuccal region (OSCC-GB) were recruited into this study with written informed consent. From each patient, a sample of tumor tissue and adjacent normal tissue were sampled by one of us (P.A.). The tissue samples were stored appropriately. TNM staging of 40 tumor samples were done following the 7th edition of the American Joint Committee on Cancer (AJCC) [11]. Summary statistics of demographic and clinical characteristics of the patients are provided in Table 1.

**Table 1:** Demographic and clinical characteristics of 40 gingivo-buccal oral squamous cell carcinoma patients included in this study

Clinical Characteristics*	Frequency	Percent
**Age**		
< 40	7	0.18
40–50	16	0.40
51–60	11	0.28
> 60	6	0.15
**Gender**		
Male	33	0.83
Female	7	0.18
**Risk-habit**		
Chewing Tobacco	19	0.48
Chewing Tobacco and (Smoking and/or Alcohol)	16	0.40
Smoking and/or Alcohol	4	0.10
None	1	0.03
**Tumor Stage**		
T1	9	0.23
T2	12	0.30
T4	19	0.48
**Lymph Node Invasion**		
N0	21	0.53
N1	10	0.25
N2	9	0.23

*All patients were M0 (no metastasis) at the first presentation when tissue samples were collected for analysis

### DNA methylation

Methylation data from paired tumour and adjacent normal tissue samples of 40 OSCC-GB patients were generated using the Illumina Infinium MethylationEPIC BeadChip [4]. Using the R package minfi, we estimated for each CpG site, the CpG-specific methylation level (β-value) as the ratio of the intensity of methylated (M) to the combined intensities of both methylated (M) and unmethylated (U) alleles:

$$ \beta =\frac{M^{\ast }}{M^{\ast }+{U}^{\ast }+C} $$

where M^*^ and U^*^ denote signal intensities of M and U alleles, respectively, and the constant C set at 100 (as recommended by the BeadChip manufacturer) [4, 12, 13]. The β-value ranges from 0 (unmethylated) to 1 (methylated). The sites that had a detection *p*-value ≥0.01 and those that mapped to X or Y chromosomes were removed. We further removed (a) probes that masked with “NA” values, (b) SNP associated probes with minor allele frequency (MAF) > 0.01, (c) probes that overlapped with a repetitive element, (d) multi-mapped probes, (e) probes that did not map to annotated protein-coding genes [4, 12, 14, 15], and (f) probes that mapped to 3’UTR region of the genome.

### Random Forest classifier

To analyze the difference between Tumor and Normal samples, a Random Forest (RF) method was used on Methylation data as implemented in the *randomForest* package in R [12, 14–19]. The random forest algorithm is an ensemble classifier similar to Classification and Regression Tree (CART) [17]. Each tree in an RF is built by choosing a bootstrap sample of two-third of the total number of individuals; the remaining one-third (Out-Of-Bag [OOB] sample) is utilised for validation. For each node in a tree, a binary splitting rule is used on a sample of CpG sites from the bootstrap sample to find the best split. The variable with the maximum information gain [20] is selected. A parameter *mtry* defines the number of variables randomly selected for each node in a tree, and another parameter *ntree* specifies the number of trees to be built in a forest. Normally, the value of *mtry* is taken to be the square root of the number of variables; this is also the default value in the R package. The output of *randomForest* provides an aggregated misclassification error (OOB error rate), which is estimated from predictions made on the OOB samples, and variable importance, which measures the weighted mean of the improvement in individual trees by each variable [15–17, 21]. The most reliable variable importance method is “permutation accuracy importance” or “Mean Decrease Accuracy” (MDA) [21, 22]. MDA permutes the data of *i* ^*th*^ variable in the OOB sample and records the permuted OOB error rate. The difference of the original and permuted OOB error rate averaged over the number the trees gives the importance score for *i* ^*th*^ variable (*VI*_*i*_) in the random forest [19, 21–23]. A high value of MDA implies greater importance of the variable [21, 22].

$$ {VI}_i=\frac{1}{ntree}\sum \limits_{j=1}^{ntree}\left({OOBerror}_{ij}^{permuted}-{OOBerror}_{ij}\right) $$

### Classification of samples

For efficient computation, only probes with |average Δβ| ≥ 0.2 were considered, where each Δβ was calculated by obtaining the difference between the β-values of tumor and adjacent normal samples of a patient for each probe indicating differential methylation between them and then taking average over the number of patients. A CpG site was considered hypermethylated if average Δβ ≥ 0.2 and hypomethylated if average Δβ ≤ − 0.2 [4]. Before implementing the random forest (RF) classifier, ntree and mtry parameters were tuned to generate an accuracy rate [12, 16]. The best performing combination of parameters were those for which the OOB error rate stabilised and reached a minimum; i.e., the combination of parameters with the highest accuracy rate. Once the optimum set of parameters was determined, “randomForest” was executed 50 times on the methylation data of 40 paired samples. In each iteration variables (probes) with MDA-score > 0 were only selected [18]. The selected probes were then mapped to their respective genes. A gene was considered for further analyses if it satisfied the following conditions: (a) there were at least two probes in the non-promoter region of the gene, (b) methylation status of all probes in the non-promoter region were unidirectional; either hypermethylated or hypomethylated, and (c) had no probes in the promoter region. The stringency of criteria (a) and (b) were adopted to minimize the chance of false-positive discovery, and the criterion (c) was adopted to make discoveries attributable to gene-body methylation only.

### RNA sequencing

RNA was extracted and RNA sequencing was performed to obtain levels of transcription of genes, on the same set of 40 paired samples. Paired-end libraries were constructed and sequenced using Illumina HiSeq2500 [4, 24]. The quality of the RNA-Seq reads was checked by FastQC. *TopHat2* [4, 24–26] was then used to align these reads to a hg19 reference transcriptome or genome. Multi-mapped reads and non-concordant reads were filtered out using *SAMtools* [4] and duplicate reads were removed using *MarkDuplicates* from PICARD [4]. *Cufflinks* [4, 24–26] was then used to assemble and reconstruct the transcriptome. Finally, using *Cuffnorm*, normalised FPKM values for each gene were estimated [4]. Only those genes that had non-zero transcription levels in all samples were considered for further analysis. We have used the level of transcription of a gene as a proxy for the level of expression of the gene, and have used transcription and expression levels interchangeably in this report.

### Integration of methylation and transcription data

Those genes for which there was no promoter probe and with multiple probes in the non-promoter region that were uniformly hyper- or hypo-methylated, and for which the level of transcription/expression change between tumour and normal tissues, averaged over the 40 pairs of samples, was higher than two-fold, were identified to be dysregulated by methylation in non-promoter regions [4]. Methylation effects on the 1st exon are similar to those of the promoter and exon boundary methylation modulates alternative splicing events [27]. Since this study is focused on gene expression alterations due to aberrant methylation on gene body, the genes that had 1st exon [28, 29] and exon boundary [27, 30] probes were removed. Finally, we considered only those genes for mapping on pathways that satisfied the known biological directionality of control; genes with hypermethylation (hypomethylation) in the gene-body region in the tumour tissue should have a significantly higher (lower) level of expression in the tumor tissue [31, 32].

### Enrichment analysis of pathways

Genes that were so identified by the integration of both methylation and expression data were analyzed for enrichment of biological pathways. We considered pathways in KEGG for this analysis. ClueGo and CluePedia plug-ins of Cytoscape were used. To identify whether a pathway in KEGG was significantly enriched, a right-sided test based on hypergeometric distribution was used. Benjamini-Hochberg correction method was used to correct the *p-*values for multiple testing [4, 33].

## Results

### Identification of genes with abundant methylation in the non-promoter region

A total of 484,420 autosomal probes with detection p-value < 0.01 were associated with 18,688 genes. After removing 3’UTR and unannotated probes, 333,208 probes remained which were associated with 18,684 genes. Of these, 22,711 probes were with |average Δβ| ≥ 0.2 that mapped to 7027 genes. By fine-tuning (Figure S1), a stable OOB error rate was obtained with default mtry = 150 and ntree = 2000. Random forest was executed 50 times, with these optimal values of the parameters. The MDA scores of each variable and OOB error rate were recorded for 50 iterations. A uniform OOB error rate of 1.25% was observed in each iteration (Table S1). The set of probes with MDA > 0 comprised 10,105 probes that mapped to 4831 genes. Among these, for 433 genes all probes in the non-promoter region were hypermethylated, and for 233 genes all were hypomethylated. We have focused on these 666 unidirectionally methylated genes, for drawing further inferences integrated with gene expression patterns in tumor-normal paired tissues.

### Integration of methylation and gene-expression

In paired tissues collected from the 40 OSCC-GB patients, non-zero levels of transcription/expression were found for 477 genes. Considering the 666 genes that exhibited significant and unidirectional methylation, it was found that 132 of these genes showed at least two-fold difference in the level of expression between tumour and normal tissues, averaged over the 40 patients. Of these 132 genes, 8 genes were removed as they had 1st exon and exon boundary probes. However, of these 124 only for 67 (54%) genes, the direction of change of expression level was consistent with that of methylation change (Table 2). That is, genes with hypermethylation (hypomethylation) in the tumour tissue had significantly higher (lower) levels of expression in the tumor tissue.

**Table 2:** Results of 67 genes that showed significant relationship between methylation in the non-promoter region and gene expression

Gene	Mean of Δβ values of probes in the non-promoter region averaged over all patients	log2 fold-change of gene-expression values averaged over all patients
ABCA3	−0.242	−2.740
ADAMTS17	−0.235	−1.418
ADCY2	− 0.282	−4.311
ADCYAP1R1	−0.231	−3.227
AFAP1L2	0.278	1.749
AGRN	0.299	1.830
ANGPT1	−0.255	−1.084
ANK2	−0.292	−3.425
ARNT2	−0.257	−1.056
ATP8A1	−0.263	−1.419
BCL11B	0.344	1.154
BMPER	−0.226	−2.030
BNC2	−0.284	− 2.276
CACNA1D	−0.261	−2.461
CACNA2D1	−0.256	−2.616
CADM1	−0.256	−1.519
CDCA7	0.235	1.265
CIT	0.274	1.201
CLIC5	−0.264	−3.547
COBL	−0.283	−3.073
COL27A1	0.323	1.918
DNAH17	0.228	3.230
EEPD1	−0.245	−1.291
EGFR	0.240	1.147
EPHB2	0.285	2.239
EPSTI1	0.269	2.851
EXT1	0.272	1.068
FAM13C	−0.340	−1.862
FAM171A1	−0.232	−1.712
FGD5	−0.266	−1.149
FHIT	−0.289	−2.022
GFI1	0.318	1.787
ICAM5	0.301	1.476
IGDCC4	−0.296	−2.178
KCNAB1	−0.279	−1.560
LAMB4	−0.206	− 1.731
LDB2	−0.268	−1.250
LRP8	0.227	1.393
MCF2L	−0.242	−2.238
MEGF11	−0.257	−1.094
NCS1	0.290	1.266
NDRG1	0.208	1.343
NKAIN1	−0.241	−1.384
NTRK3	−0.298	−3.202
PALM	−0.274	−2.500
PAPPA	0.258	1.324
PARK2	−0.278	−2.854
PDZRN3	−0.250	−1.409
PLCL1	−0.243	−1.626
PML	0.220	1.562
PPM1L	−0.303	−2.386
PRKD1	−0.276	−1.051
RGS20	0.221	2.582
RTKN	0.235	1.183
SCIN	−0.278	−3.784
SDK2	0.247	2.410
SLC6A17	−0.240	−1.586
SLC7A5	0.241	1.367
SOBP	−0.286	−2.687
SPRED3	0.280	1.989
SUSD4	−0.319	−1.780
TECTA	−0.286	−1.007
TENM2	0.275	2.951
TMEM232	−0.213	−1.995
TRAM2	0.264	1.327
WNK2	−0.217	−3.895
ZNF423	−0.254	−1.799

### Enriched pathway

The pathway enrichment analysis using the 67 genes dysregulated by methylation alteration in the gene-body region between tumour and normal tissues, identified enrichment of one significant (corrected *p*-value = 0.0012) KEGG pathway. This was Central Carbon metabolism in Cancer with three associated genes EGFR, NTRK3, and SLC7A5. It has been reported, based on cell line studies, that overexpression of EGFR can impact on the development of solid tumors, including oral cancer [34]. It was found that EGFR was overexpressed and globally hypermethylated.

## Discussion

**Fig. 1:**
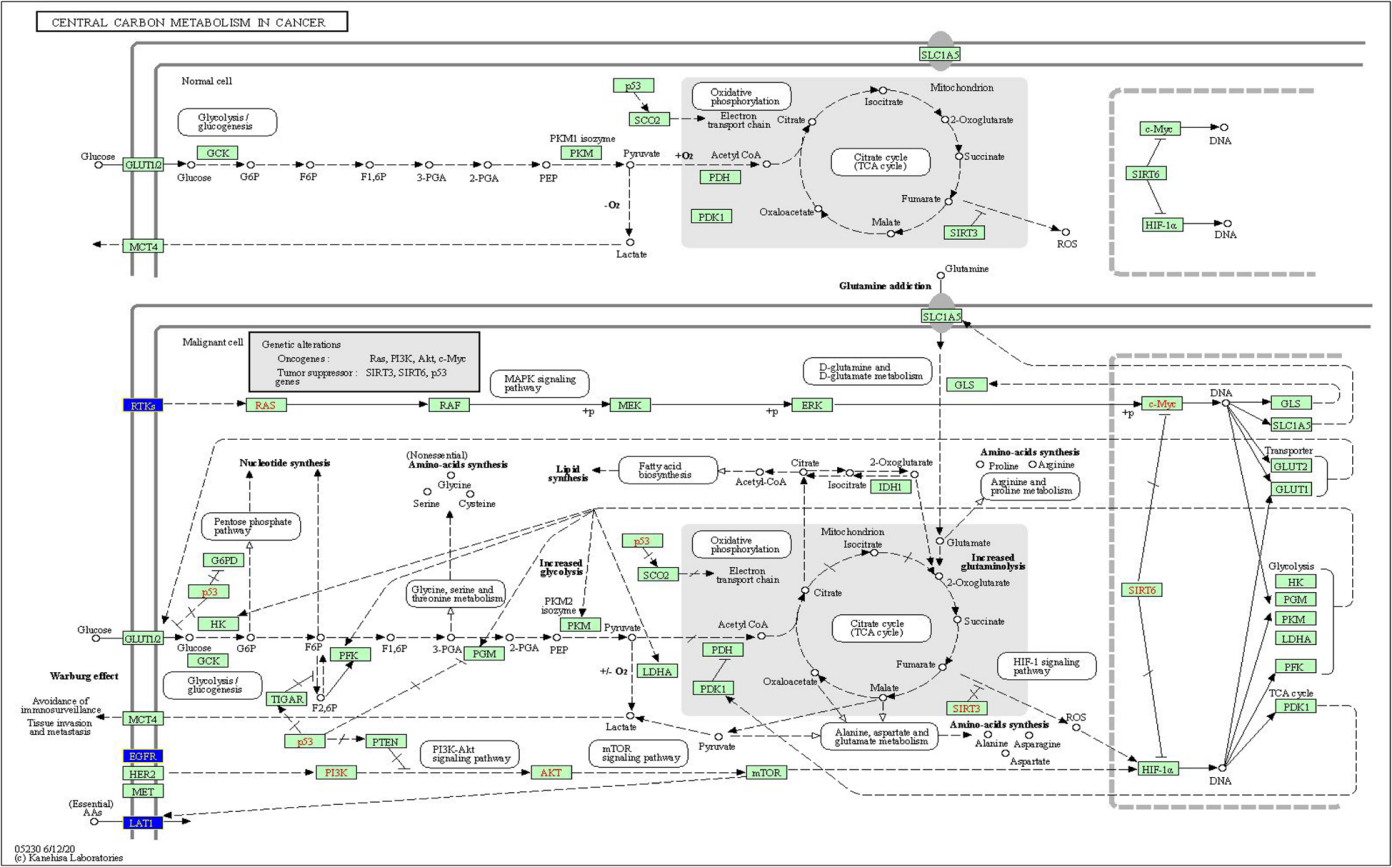
Genes found altered in Central Carbon Metabolism (CCM) pathway in gingivo-buccal oral cancer. EGFR, SLC7A5, NTRK3, the three key genes (marked in blue and appearing on the vertical lines to the left of the figure), were significantly dysregulated in the CCM pathway

By applying the novel Random Forest data-adaptive method to high-dimensional data (about 500,000 data points per individual) to identify significant alterations in gene-body methylation in gingivo-buccal oral tumor tissue compared to adjacent normal tissue, and subsequent integration with gene expression data it was detected that some genes and pathways were not earlier inferred to be involved in OSCC-GB only through cell-line studies. Although we found that only about 54% of genes found to have aberrant methylation were also dysregulated in the expected direction, this is not unexpected because gene-body methylation may not be the only cause of dysregulation of a gene. Hence, the directionality of dysregulation may not be in accord with what is expected under the methylation-transcription model. As a matter of fact, it is striking that over 50% of genes show transcription levels in accord with what is expected under gene-body hyper- or hypo-methylation. The significantly enriched pathway that has been identified using this data-adaptive and data-integrative approach is the Central Carbon Metabolism (CCM) pathway, which is involved in transport and oxidation of main carbon sources inside the cell. Fundamental cellular processes require energy for growth. The catabolic and anabolic reactions in metabolism are finely balanced and tightly regulated. Dysregulation results in cellular transformation and tumor progression. In rapidly dividing cancer cells, metabolic reprogramming from normal cells takes place to enable enhanced proliferation. CCM pathway plays a fundamental role in metabolic reprogramming. Changes in central carbon metabolism of cancer stem cells have also been noted [35]. It is noteworthy that enrichment of the CCM pathway in OSCC-GB takes place by gene-body methylation mediated dysregulation of three key genes, EGFR, NTRK3, SLC7A5 (Fig. 1).

Significant downregulation of NTRK3 mediated by promoter methylation was noted in our earlier study [4]. NTRK3 is a neurotrophin receptor. It behaves as an oncogene in breast cancer [36, 37] and possibly also in hepatocellular carcinoma [38]. However, it also plays a dual function. It acts as a tumor suppressor in colorectal cancer in which it is epigenetically inactivated [39]. In OSCC-GB also, NTRK3 is epigenetically dysregulated and appears to behave as a tumor suppressor.

SLC7A5 – earlier known as LAT1 – is a transporter dedicated to essential amino acids. Cancer cells have an increased demand for nutrients that include glucose and essential amino acids; the so-called “Warburg effect.” Overexpression of SLC7A5, as we have observed here, is explained in part by the presence, in its promoter, of a canonical binding site for the proto-oncogene c-Myc [40] that is known to regulate glucose metabolism [41]. Overexpression of SLC7A5 is also controlled by methylation in the promoter [4] and non-promoter regions (this study).

EGFR has been earlier implicated in progression, recurrence, and stemness of oral cancer [42, 43]. EGFR is inappropriately activated in cancer mainly because of amplification and point mutations. Transcriptional upregulation of EGFR due to autocrine/paracrine mechanisms has also been described [44]. Here, for the first time, it is shown that dysregulation of EGFR takes place by epigenetic mechanisms in oral cancer.

Cancer cells rapidly multiply. Significant metabolic changes occur during cancer development and progression. Cancer cells have a lot of metabolic requirement for the increase of their biomass and genome. These include the increased demand for nutrients such as glucose, essential amino acids and also glutamine, that becomes conditionally essential, for protein synthesis and/or energy supply [45–48]. Cancer cells utilize large amounts of glucose and glutamine and maintain high rates of glycolysis and glutaminolysis; called the Warburg effect. These increased requirements are met by the cancer cells themselves. Cancer cells undergo a large number of mutations, some of which take place in genes that belong to specific pathways, such as the central carbon metabolism pathway, which help meet these additional requirements. The central carbon metabolism pathway is large, complex and performs a variety of functions. About 70 genes are involved in this pathway, that are involved in a variety of functions that include glucose import, glycolysis, pentose phosphate flux, lactate excretion, pyruvate dehydrogenase flux, TCA cycle flux, pyruvate carboxylase flux, gluconeogenic flux, glycine biosynthesis, glutathione biosynthesis, proline biosynthesis, palmitate biosynthesis (fatty acid synthase activity), desaturation of palmitate, elongation of palmitate, and desaturation of stearate [49]. Changes in one or more components of the central carbon metabolism pathway have been identified in various cancers. The genes that we have found to be significantly altered in their levels of expression resulting from gene-body methylation changes – NTRK3, SLC7A5 (LAT1) and EGFR – belong to the subcomponents related to the Warburg effect, notably glucose import and glycolysis.

## Conclusions

Three key genes NTRK3, SLC7A5 (LAT1) and EGFR were dysregulated in the CCM pathway. Of these, NTRK3 [4, 50] and SLC7A5 [4] were earlier identified to be associated with oral cancer. However, we provide the first evidence of epigenetic impact on overexpression of EGFR in oral cancer. To enable enhanced proliferation of cells in a cancer tissue, metabolic reprogramming from normal cells usually takes place. In the present analysis, we have identified that among oral cancer patients, genes in the CCM pathway – that plays a fundamental role in metabolic reprogramming – are significantly dysregulated because of perturbation of methylation in non-promoter regions of the genome. This result compliments our previous result that perturbation of promoter methylation results in significant changes in key genes that regulate the feedback process of DNA methylation for the maintenance of normal cell division. Taken together, it is evident that in oral cancer methylation driven alterations in both promoter and non-promoter genomic regions result in disruption of normal cell division accompanied by metabolic reprogramming to enable rapid cell proliferation.

## Supplementary Information

**Additional file 1: Figure S1.** Fine-tuning *randomForest* parameters *mtry* and *ntree*.

**Additional file 2: Table S1.** Results of 50 iterations of tuned Random Forest classifier showing the number of variables selected and the OOB error rate.

## Data Availability

Raw IDAT files of 40 samples generated using Illumina Infinium methylation array were deposited under EGAS00001003896 EGA study ID and aligned bam files for transcriptome data of 40 samples were deposited under EGAS00001003893 EGA study ID. Biospecimens may be shared on request, if not exhausted. Dispatch of biospecimens requires prior approval from the Government of India.

## Abbreviations

RF: Random Forest
OSCC-GB: Oral Squamous Cell Carcinoma of the Gingivo Buccal region
CCM: Central Carbon Metabolism pathway
MAF: Minor Allele Frequency
CART: Classification And Regression Trees
OOB: Out-Of-Bag sample
MDA: Mean Decrease Accuracy
VI: Variable Importance
FPKM: Fragments Per Kilobase of transcript per Million mapped reads
KEGG: Kyoto Encyclopedia of Genes and Genomes
