# Supplementary material for: Application of Random Forest and data integration identifies three dysregulated genes and enrichment of Central Carbon Metabolism pathway in Oral Cancer
Source: BMC Cancer. 2020 Dec 14;20:1219. doi: 10.1186/s12885-020-07709-0 (PMC7737291; doi:10.1186/s12885-020-07709-0)
Supplement: Supplementary file 1 — Additional file 1: Figure S1. Fine-tuning randomForest parameters mtry and ntree. [file 12885_2020_7709_MOESM1_ESM.pdf]

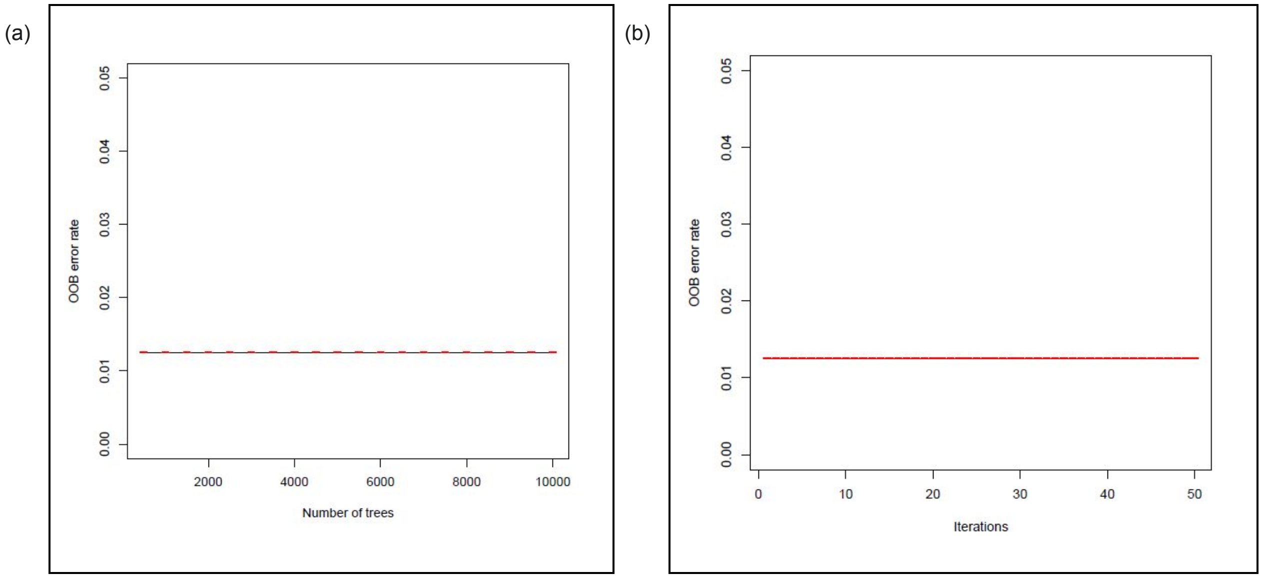

Figure S1. Fine-tuning *randomForest* parameters *mtry* and *ntree*.

(a) Tuning *ntree* using R package "*tuneNTREE*" with values starting from 500 to 10,000. Each *ntree* value was iterated 5 times and obtained a 1.25% error rate. Error bar is displaying the standard deviation of the OOB error rate for each *ntree*. (b) Tuning *mtry* using R package "*tuneRF*" with mtry values ½√p, √p (default value=150) and 2√p, where p is the number of variables = 22,711 and ntree value 2000 for each 50 iterations. The error rate for ½√p, √p and 2√p for each iteration was 1.25%. Error bar is displaying the standard deviation of OOB error rate for different *mtry* in each iteration.
