# Supplementary material for: Application of Random Forest and data integration identifies three dysregulated genes and enrichment of Central Carbon Metabolism pathway in Oral Cancer
Source: BMC Cancer. 2020 Dec 14;20:1219. doi: 10.1186/s12885-020-07709-0 (PMC7737291; doi:10.1186/s12885-020-07709-0)
Supplement: Supplementary file 2 — Additional file 2: Table S1. Results of 50 iterations of tuned Random Forest classifier showing the number of variables selected and the OOB error rate. [file 12885_2020_7709_MOESM2_ESM.pdf]

Table S1. Results of 50 iterations of tuned Random Forest classifier showing the number of variables selected and the OOB error rate.

Iteration number	No. of variables selected (MDA>0)	OOB error rate (%)	Iteration number	No. of variables selected (MDA>0)	OOB error rate (%)
1	1349	1.25	26	1361	1.25
2	1334	1.25	27	1337	1.25
3	1331	1.25	28	1329	1.25
4	1329	1.25	29	1325	1.25
5	1329	1.25	30	1360	1.25
6	1389	1.25	31	1370	1.25
7	1353	1.25	32	1363	1.25
8	1312	1.25	33	1355	1.25
9	1348	1.25	34	1363	1.25
10	1359	1.25	35	1328	1.25
11	1353	1.25	36	1311	1.25
12	1335	1.25	37	1354	1.25
13	1344	1.25	38	1325	1.25
14	1368	1.25	39	1367	1.25
15	1316	1.25	40	1366	1.25
16	1351	1.25	41	1401	1.25
17	1329	1.25	42	1341	1.25
18	1323	1.25	43	1341	1.25
19	1347	1.25	44	1374	1.25
20	1390	1.25	45	1337	1.25
21	1365	1.25	46	1338	1.25
22	1392	1.25	47	1372	1.25
23	1385	1.25	48	1361	1.25
24	1363	1.25	49	1343	1.25

25	1355	1.25	50	1332	1.25
